# Bouveret Syndrome Mimicking Gallbladder Malignancy: A Diagnostic Challenge in an 85-Year-Old Woman

**DOI:** 10.7759/cureus.116109

**Published:** 2026-09-11

**Authors:** Dominik Megias, Beat Oertli, Peter Bauerfeind

**Affiliations:** 1 Internal Medicine, Hirslanden Klinik St. Anna, Lucerne, CHE; 2 Gastroenterology and Hepatology, Hirslanden Klinik St. Anna, Lucerne, CHE

**Keywords:** bouveret syndrome, cholecystoduodenal fistula, gallbladder neoplasm, gallstone ileus, gastric outlet obstruction, tumor mimic

## Abstract

Bouveret syndrome is a rare complication of cholelithiasis in which a gallstone enters the duodenum via a cholecystoenteric fistula, causing gastric outlet obstruction. Because of chronic inflammation and nonspecific symptoms, the condition can easily be misdiagnosed as a gastrointestinal malignancy. An 85-year-old woman presented with severe weight loss (10 kg), night sweats, and non-bloody vomiting. CT imaging revealed massive aerobilia, a thickened gallbladder wall, and an ill-defined mass at the gastroduodenal junction. These findings raised strong suspicion for a malignant process in the gallbladder or pancreatic head with gastroduodenal infiltration. However, subsequent upper endoscopy revealed the mass to be a huge impacted gallstone obstructing the duodenal bulb. The calculus was successfully fragmented and removed using intracorporeal electrohydraulic lithotripsy, which generates shock waves, resulting in complete resolution of the obstruction without high-risk surgery. Bouveret syndrome can closely mimic the clinical and radiological presentation of gastrointestinal malignancies. Physicians must maintain a high index of suspicion for this syndrome in elderly patients presenting with gastric outlet obstruction with features suggestive of malignancy and aerobilia, as prompt endoscopic evaluation can spare them from unnecessary oncologic interventions.

## Introduction

Bonnet first described gastric outlet obstruction caused by large gallstones at autopsy in 1841, and Leon August Bouveret established the first preoperative diagnosis in 1896. Mechanical obstruction of this nature occurs in only 0.3-0.5% of patients with cholelithiasis [1]. It typically results from a gallstone passing through a cholecystoenteric fistula, which forms secondary to chronic inflammation, adhesions, pressure necrosis from large calculi, and subsequent wall ischemia.

While most migrating stones pass without issue, stones larger than 2 cm can become impacted, most commonly in the terminal ileum. In 1-3% of these cases, however, the stone obstructs the duodenum, a condition known as Bouveret syndrome. Diagnosis is notoriously challenging due to the nonspecific nature of the symptoms, which typically include epigastric pain, nausea, vomiting, and weight loss [2,3].

A major diagnostic pitfall is that Bouveret syndrome can closely mimic gastrointestinal malignancies. Chronic inflammation and stricture formation can simulate neoplastic infiltration on imaging, and approximately 25% of gallstones are isoattenuating and missed on standard CT [3,4]. Furthermore, patients with biliary-enteric fistulas have a higher reported incidence of gallbladder cancer (3-14%) compared to those with uncomplicated cholelithiasis (0.3%) [5]. This report highlights a case where Bouveret syndrome masqueraded clinically and radiologically as a malignant tumor, emphasizing the critical role of endoscopy in establishing the correct diagnosis.

## Case presentation

An 85-year-old woman presented to the emergency department with a six-day history of non-bloody vomiting, diarrhea, and general weakness. Crucially, her medical history over the past year was dominated by severe "B symptoms": an unintentional weight loss of approximately 10 kg accompanied by profound night sweats. She denied any abdominal pain, fever, or recent travel. Her medical history included arterial hypertension managed with nebivolol, type 2 diabetes mellitus treated with gliclazide, and essential thrombocythemia managed with aspirin and hydroxycarbamide. On physical examination, she weighed 44 kg, was 157 cm tall, and was hemodynamically stable. Her abdomen was soft without tenderness or palpable masses. Initial laboratory results showed mild thrombocytosis (391 G/l) and elevated serum urea (17 mmol/L), but liver enzymes, cholestasis parameters, and inflammatory markers (CRP 15 mg/l, procalcitonin 0.10 ng/mL) were only mildly elevated (Table 1).

**Table 1:** Laboratory findings at admission CRP: C-reactive protein

Parameter	Admission	Reference range
Hemoglobin	126 g/L	126-160 g/L
Leukocytes	6.3 G/L	4.0-10.0 G/L
Platelets	391 G/L	150-350 G/L
Urea	17 mmol/L	2.8-8.1 mmol/L
CRP	15 mg/L	<5.0 mg/L
Procalcitonin	0.1 ng/mL	0.05-0.1 ng/mL
Creatinine	78 µmol/L	45-84 µmol/L
Sodium	141 mmol/L	136-145 mmol/L
Potassium	3.8 mmol/L	3.50-5.10 mmol/L
Phosphate	0.97 mmol/L	0.87-1.45 mmol/L
Corrected calcium	2.54 mmol/L	2.20-2.55 mmol/L

Given the alarming history of severe weight loss and night sweats, a thoracoabdominal CT was performed specifically to screen for a suspected malignancy. The CT demonstrated massive aerobilia, dilated central biliary ducts (maximum 10 mm), and a dilated pancreatic duct. The gallbladder was irregularly configured with a thickened wall and surrounding imbibition. An ill-defined mass was identified at the duodenal papilla, causing massive gastric retention (Figure 1). These findings strongly suggested a malignant process in the gallbladder or pancreatic head with gastroduodenal infiltration.

**Figure 1:**
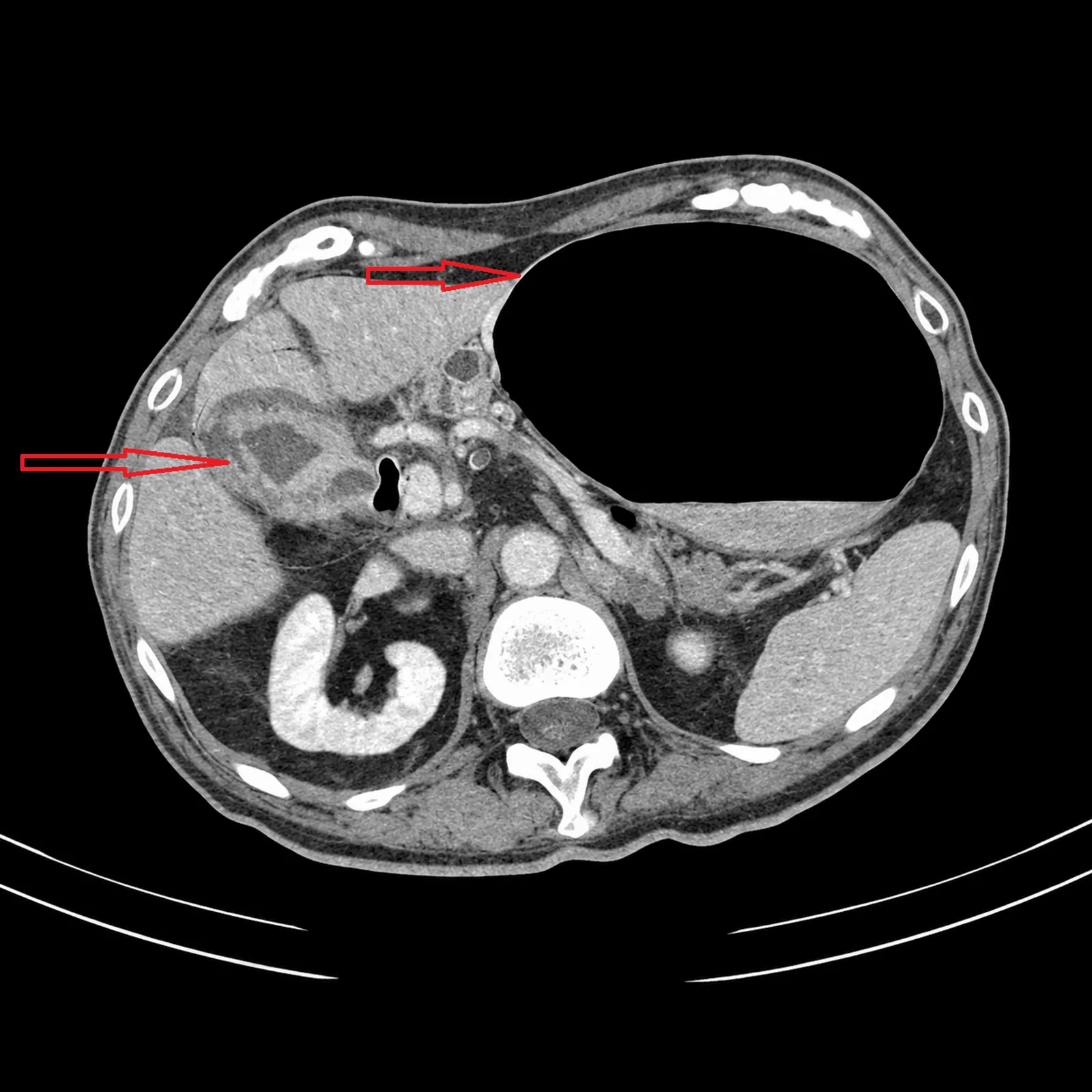
Contrast-enhanced CT of the abdomen demonstrating an ill-defined duodenobiliary mass and a distended retention stomach (red arrows) CT: computed tomography

To further investigate the suspected malignancy, we performed an urgent upper endoscopy. Surprisingly, the endoscopy revealed a severe obstruction of the duodenal bulb caused by a very large impacted biliary stone rather than a tumor, but by a very large impacted biliary stone. We successfully fragmented the stone using electrohydraulic lithotripsy over two sessions. The extracted stone fragments collectively measured 6 × 3 × 2 cm in total (Figure 2). We initiated prophylactic antibiotic therapy with ceftriaxone. After stone clearance, an endoscopic retrograde cholangiopancreatography confirmed a patent cholecystoduodenal fistula, and a Spaxus stent was placed to maintain biliary drainage. The patient recovered well, began gaining weight via a transluminal gastric tube, and was discharged home in much better condition.

**Figure 2:**
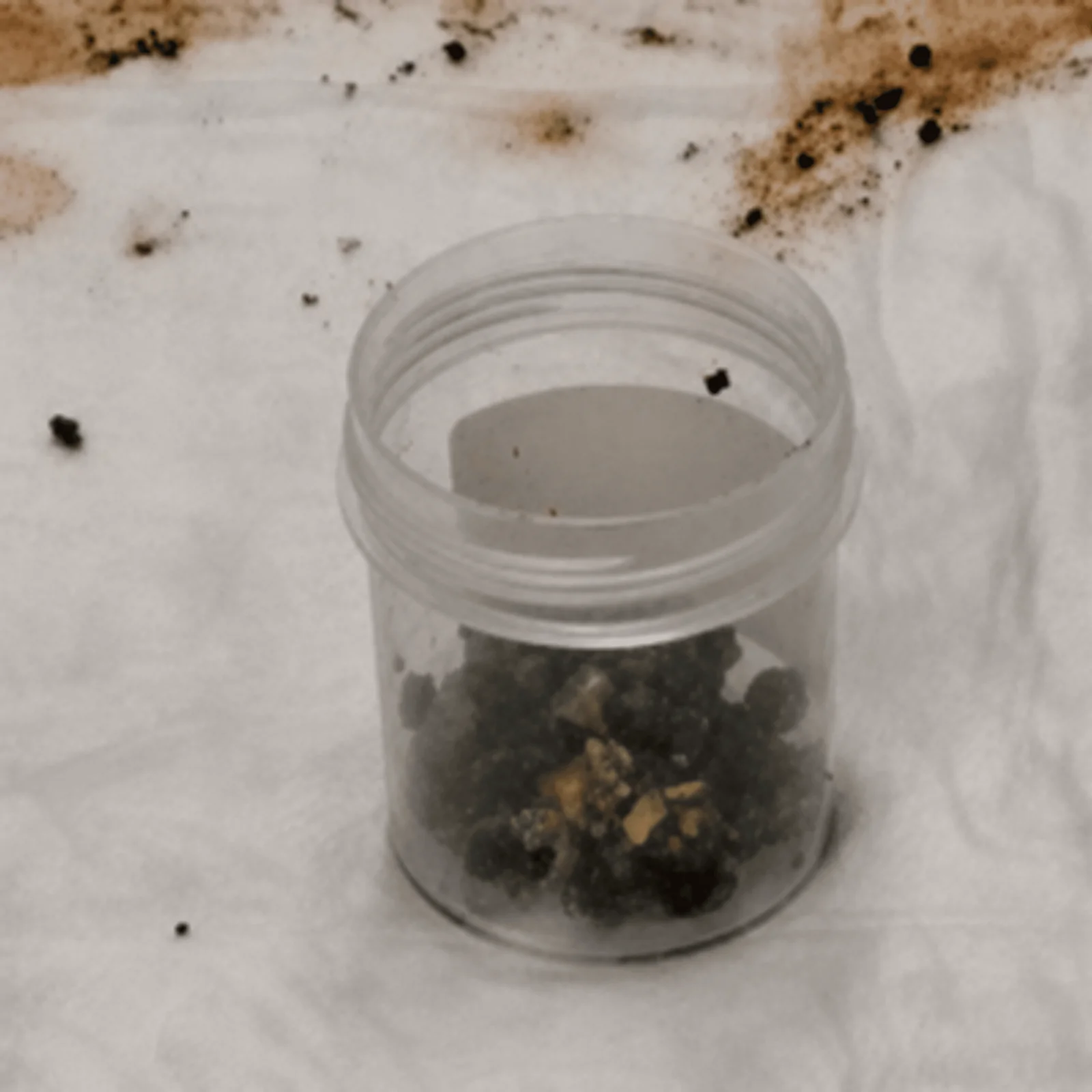
Successful endoscopic extraction of multiple calculus fragments following lithotripsy was achieved

The Spaxus stent was removed two weeks after the initial intervention without complications. During subsequent follow-up with her primary care physician, she showed steady clinical improvement. Given the patient's advanced age and multiple comorbidities, routine follow-up imaging, such as magnetic resonance cholangiopancreatography, for malignancy surveillance was not pursued. At present, four years after her initial hospital presentation, the patient remains alive and clinically well.

## Discussion

The most striking feature of this case was the profound overlap in clinical and radiological presentation between Bouveret syndrome and a gastrointestinal malignancy. The patient's chronic weight loss (10 kg) and night sweats strongly suggested a neoplastic process [6,7]. However, these "B symptoms" were ultimately the result of a chronic, slowly progressing gastric outlet obstruction that led to severe malnutrition and dehydration. Although the night sweats cannot be fully explained by the findings described above, they did not recur during the subsequent course. We therefore suspect that they may nevertheless have been related to the underlying condition. The visible calcification within the obstructing mass required consideration of other calcified subepithelial neoplasms of the gastroduodenal tract, such as gastrointestinal stromal tumors or leiomyomas, which can occasionally show similar attenuation on imaging. This diagnostic challenge underscores CT's inherent limitations in reliably differentiating Bouveret syndrome from other pathologies [7,8].

CT limitations can hinder the radiological diagnosis of Bouveret syndrome. While Rigler’s triad (bowel obstruction, pneumobilia, and an ectopic gallstone) is classic, nearly 25% of gallstones cannot be seen on CT due to bile/fluid isoattenuation [2,8]. In our patient, the irregularly configured, thickened gallbladder wall with surrounding inflammatory changes, combined with a mass at the papilla and a dilated pancreatic duct, perfectly simulated the radiological appearance of a pancreatic head or gallbladder carcinoma infiltrating the duodenum [9].

In such cases, initial suspicion of malignancy is not merely a diagnostic confounder but a legitimate clinical concern. The pathophysiological process that creates a cholecystoenteric fistula (chronic inflammation, pressure necrosis, and continuous exposure of the biliary system to gut flora and chemical irritation) can trigger epithelial cell changes. As a result, large studies report a significantly higher rate of gallbladder cancer in patients with biliary-enteric fistulas (3-14%) compared with patients with uncomplicated cholelithiasis (0.3%) [1]. Therefore, even when Bouveret syndrome is confirmed endoscopically, physicians must remain vigilant. If single-stage surgical management involving cholecystectomy is not performed, as was the case for our patient, who was treated purely endoscopically, ongoing clinical and imaging follow-up is recommended to monitor for the potential development of a gallbladder mass.

Therapeutically, endoscopic electrohydraulic lithotripsy proved to be a highly effective, minimally invasive option for this frail 85-year-old patient [10]. Avoiding a high-risk laparotomy was crucial, as surgical interventions for gallstone ileus carry significant morbidity and a reported mortality rate of up to 25%. More recent data suggest a mortality rate slightly above 10% [11].

## Conclusions

Bouveret syndrome is a rare condition that can closely mimic a gastrointestinal malignancy, presenting with severe weight loss, night sweats, and obstructive masses on imaging. Clinicians must maintain a high index of suspicion for this syndrome when evaluating elderly patients with gastric outlet obstruction and aerobilia. Because chronic biliary-enteric fistulas are also associated with an increased risk of concurrent gallbladder carcinoma, prompt endoscopic evaluation is vital to differentiate an impacted stone from a true neoplasm and to guide safe, minimally invasive therapy.
